# Safety and Cost‐Effectiveness of Early Discharge Following Bariatric Surgery in an Underserved Urban Population

**DOI:** 10.1155/jobe/4162764

**Published:** 2026-02-06

**Authors:** Dimitri Chepkunov, Daye Chung, Oyinem Odumah, Leaque Ahmed, Paritosh Suman

**Affiliations:** ^1^ School of Medicine, St. George’s University, True Blue, Grenada, sgu.edu; ^2^ Department of General Surgery, Wyckoff Heights Medical Center, Brooklyn, New York, USA, wyckoffhospital.org

## Abstract

This study evaluated the safety and economic impact of discharging bariatric surgery patients on Postoperative day (POD) 1 compared to the institutional POD 2 discharge protocol. A retrospective review of 115 patients who underwent laparoscopic or robot‐assisted sleeve gastrectomy and Roux‐en‐Y gastric bypass (RYGB) revealed no significant differences in complications or readmissions between the groups. Discharge on POD 1 demonstrated a cost difference of approximately $1571. Our findings support the safety, feasibility, and financial benefits of implementing POD 1 discharge as standard practice for bariatric patients in underserved urban populations.

## 1. Introduction

Bariatric surgery is widely recognized as one of the most effective interventions for the treatment of severe obesity, offering significant weight reduction, improvement of obesity‐related comorbidities, and enhanced quality of life [1]. Commonly performed procedures include sleeve gastrectomy, Roux‐en‐Y gastric bypass (RYGB), adjustable gastric banding, and biliopancreatic diversion with duodenal switch, each differing in complexity, efficacy, and risk profile.

Despite its proven benefits, bariatric surgery requires meticulous perioperative management to optimize outcomes and minimize complications. Postoperative recovery includes several key elements, including adequate pain control, hydration, nutritional management, early mobilization, and vigilant monitoring for complications. Traditionally, patients are admitted for observation for at least 48 h postoperatively, during which clinical teams monitor vital signs, manage pain and nausea, guide dietary progression from clear liquids to full liquids, and encourage frequent ambulation.

Common early postoperative complications include nausea, vomiting, dehydration, infection, venous thromboembolism, anastomotic leaks, bleeding, and respiratory difficulties such as atelectasis or pneumonia. Long‐term risks may include nutritional deficiencies, strictures, bowel obstruction, and dumping syndrome, particularly after gastric bypass procedures.

Reducing hospital length of stay, while maintaining patient safety and satisfaction, has increasingly become a major priority in healthcare delivery, especially amid rising healthcare costs and limited resources. Advances in minimally invasive surgical techniques, anesthesia practices, Enhanced Recovery After Surgery (ERAS) protocols, and remote monitoring have enabled clinicians′ consideration of early discharge strategies without compromising patient safety [1, 2].

The primary objective of this study was to evaluate the safety, feasibility, and cost‐effectiveness of Postoperative day (POD) 1 discharge following bariatric surgery in an underserved urban population. By evaluating clinical outcomes, we aim to support the adoption of early discharge protocols in resource‐limited settings while maintaining optimum quality, safety, and cost‐effective standards.

## 2. Materials and Methods

Following institutional review board (IRB) approval, a retrospective chart review was conducted of 115 adult patients who underwent laparoscopic or robot‐assisted sleeve gastrectomy and gastric bypass at a single urban safety net hospital between January 2023 and October 2024. Patients were stratified into two groups based on their discharge day: POD 1 (*n* = 36) and POD 2 (*n* = 79), the institution’s standard discharge day.

**Figure 1:**
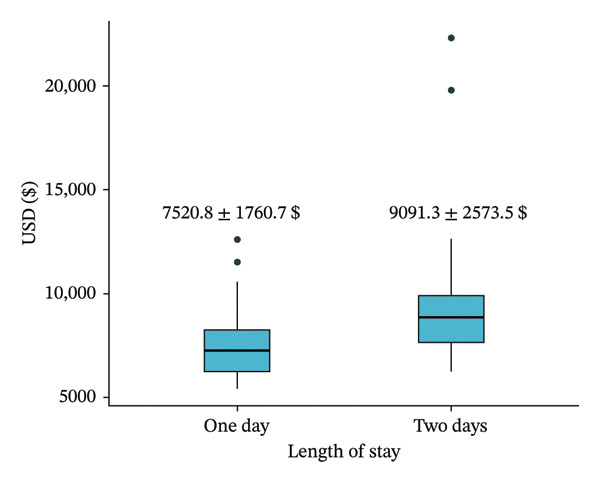
Cost analysis between POD 1 and POD 2 discharge groups.

Data collected included patient demographics (age, sex, body mass index [BMI], and comorbidities), operative details (operative time, surgical approach, and estimated blood loss), and perioperative outcomes (Table 1). Primary clinical endpoints were complication and readmission rates. Complications were defined as any adverse clinical event requiring medical or surgical intervention occurring during the study period. Cost analysis was performed, including operating room (OR) time and average daily hospital stay costs (Table 2 and Figure 1).

**Table 1:** Baseline characteristics and comorbidities of patients discharged on POD 1 vs. POD 2.

Variable	LOS		Total (*n* = 115)	*p* value
	One day (*n* = 36)	Two days (*n* = 79)		
Age	39.53 ± 13.36	39.05 ± 12.4	39.2 ± 12.65	0.853
BMI	41.85 ± 4.64	44.256 ± 8.1	43.503 ± 7.2	0.099
Preop hemoglobin	13.1 ± 1.1	13.316 ± 1.64	13.24 ± 1.5	0.435
Sex				0.346
Female	30 (83.3%)	59 (74.7%)	89 (77.4%)	
Male	6 (16.7%)	20 (25.3%)	26 (22.6%)	
Smoking				0.067
Never	30 (83.3%)	68 (86.1%)	98 (85.2%)	
Current	6 (16.7%)	5 (6.3%)	11 (9.6%)	
Former	0 (0.0%)	6 (7.6%)	6 (5.2%)	
Diabetes	8 (22.2%)	32 (40.5%)	40 (34.8%)	0.061
Hypertension	13 (36.1%)	30 (38.0%)	43 (37.4%)	0.848
Thyroid disease	6 (16.7%)	8 (10.1%)	14 (12.2%)	0.323
Anticoagulation	3 (8.3%)	10 (12.7%)	13 (11.3%)	0.752
Peripheral artery disease	0 (0.0%)	2 (2.5%)	2 (1.7%)	0.336
Coronary artery disease	3 (8.3%)	1 (1.3%)	4 (3.5%)	0.09
Hyperlipidemia	8 (22.2%)	22 (27.8%)	30 (26.1%)	0.649
COPD/ASA	8 (22.2%)	26 (32.9%)	34 (29.6%)	0.278

**Table 2:** Operative metrics, postoperative outcomes, and cost analysis.

Variable	LOS		Total (*n* = 115)	*p* value
	One day (*n* = 36)	Two days (*n* = 79)		
Surgical time (min)	104.5 ± 47.01	121.9 ± 40.4	116.2 ± 43.3	0.056
EBL (mL)	48.9 ± 19.9	46.54 ± 25.8	47.3 ± 24	0.63
Readmission	0 (0.0%)	2 (2.5%)	2 (1.7%)	0.336
Reoperation	0 (0.0%)	0 (0.0%)	0 (0.0%)	> 0.999
Follow‐up (month)	2.1 ± 2.5	5.5 ± 4.5	4.453 ± 4.3	< 0.001
Cost (USD)	7520.8 ± 1760.7	9091.3 ± 2573.5	8573 ± 2442.9	< 0.001

Comparative analyses were conducted between the two groups to assess differences in clinical outcomes and economic impact associated with early discharge.

## 3. Results

Of the 115 patients included in the study, 36 were discharged on POD 1 and 79 on POD 2. Baseline demographic characteristics such as age, BMI, sex distribution, preoperative hemoglobin, and comorbidities were comparable between the two groups (Table 1). Operative metrics, including mean estimated blood loss (47.3 mL) and average surgical time (116 min), were not statistically significant between the two groups (Table 2). There were no differences in reoperation rates (0% for both) or readmission rates (0% for POD 1 and 2.5% for POD 2; *p* = 0.336) between the groups. Overall complication rates were 0% in both groups. Early discharge resulted in a significant cost difference of approximately $1571 between the groups (95% CI: $583.2–2557.8).

## 4. Discussion

In this study, early discharge on POD 1 following sleeve gastrectomy and RYGB did not demonstrate increased complication or readmission rates compared to the standard 2‐day hospital stay. These findings are consistent with a growing body of literature supporting early discharge protocols when appropriate perioperative care and close patient monitoring practices are in place [2]. Our results further highlight that shortened hospital stays when coupled with proper safeguards do not inherently compromise patient outcomes.

These inpatient safeguards generally followed ERAS protocols. Patients were on continuous postoperative vital sign monitoring for 24 h. On POD 0, aggressive early ambulation and incentive spirometry use 10 times an hour were encouraged and supervised. Maintenance intravenous fluids were administered at a rate of 150 cc per hour for 24 h; this was reduced to 75 cc per hour once the patient was tolerating a bariatric full liquid diet. Sequential compression devices and pharmacologic prophylaxis for deep venous thrombosis (DVT) were used, consisting of either subcutaneous heparin every 8 hours or enoxaparin twice daily, with regimen selection guided by patient BMI. On POD 1, laboratory values, including complete blood count (CBC), comprehensive metabolic panel (CMP), serum magnesium, and phosphorus, were obtained. If the white blood cell count was 14 or above, a repeat CBC was performed to ensure it was reactive and not pathologic. If a patient had a repeat value greater than or equal to 14 and/or abnormal vital signs, they would be kept in the hospital, and the white blood cell count would be repeated the following day. Any necessary diagnostic imaging was performed if required. Electrolytes were repleted intravenously if the potassium was below 4 mEq/L, magnesium below 2, or phosphorus below 3. A routine upper gastrointestinal (UGI) series was performed on POD 1. Diets were advanced to bariatric full liquids if the UGI was negative for complications. If patients tolerated their diet, had adequate pain control, participated in incentive spirometry and ambulation, and demonstrated normal vital signs and laboratory results, they were deemed to meet the discharge criteria. These patients were discharged home. These measures ensured patient safety before discharge and contributed to the successful outcomes observed.

A key finding in our analysis was the significant cost savings associated with early discharge; a $1571 cost difference between the two groups, underlining its economic advantages and potential for resource optimization within healthcare systems. Strategies that maintain patient safety while simultaneously reducing expenditures are invaluable in the midst of rising healthcare costs and limited inpatient capacity [3, 4]. Recent ERAS Society guidelines support shortened length of stay following bariatric surgery when standardized perioperative pathways and appropriate patient selection are employed [5].

The successful implementation of remote patient monitoring in our cohort played a pivotal role in ensuring safe early discharge. Patients discharged on POD 1 were monitored at home through digital systems tracking vital signs, oxygen saturation, weight, temperature, and blood glucose levels. These data were reviewed in real time by supervising physicians and nurses, allowing for early identification and management of any emerging issues. Ten days after discharge, all patients had their first postoperative tele‐visit to assess their pain levels, diet and medication intake, and ambulation. This system enhanced both provider and patient confidence and satisfaction, facilitating smooth recovery in the comfort of the patient’s home [1, 2].

While our findings strongly support early discharge protocols, there are some limitations that must be acknowledged. The retrospective nature of this study and the single‐institution setting may limit generalizability. Furthermore, the study only included patients undergoing sleeve gastrectomy and RYGB; thus, caution should be exercised in extrapolating these findings to other procedures such as single anastomosis duodeno–ileal bypass with sleeve gastrectomy (SADI‐S).

Further research should include larger, prospective, multicenter studies to validate these findings and help delineate which patient subsets would benefit most from shortened hospital stays. Inclusion of patients undergoing a broader range of procedures would enhance the applicability and robustness of future analyses and further support evidence‐based guidelines for perioperative management.

## 5. Conclusions

Our study reinforces the safety, feasibility, and cost‐effectiveness of discharging bariatric surgery patients on POD 1. Early discharge, when supported by appropriate perioperative protocols and remote patient monitoring, can enhance patient outcomes, reduce healthcare costs, and optimize resource utilization. Nationwide implementation of early discharge protocols may promote greater efficiency in bariatric care delivery, as well as enhanced patient and healthcare outcomes.

## Funding

The research did not receive any specific funding but was performed as part of the employment of the authors at Wyckoff Heights Medical Center, Brooklyn, NY.

## Conflicts of Interest

The authors declare no conflicts of interest.

## Data Availability

The data that support the findings of this study are available on request from the corresponding author. The data are not publicly available due to privacy or ethical restrictions.
